# VaryBlock: A Novel Approach for Object Detection in Remote Sensed Images

**DOI:** 10.3390/s19235284

**Published:** 2019-11-30

**Authors:** Heng Zhang, Jiayu Wu, Yanli Liu, Jia Yu

**Affiliations:** School of Information Engineering, East China Jiaotong University, Nanchang 330013, China; 2017068085211002@ecjtu.edu.cn (J.W.); 2612@ecjtu.edu.cn (J.Y.)

**Keywords:** remote sensing, object detection, YOLO, VaryBlock

## Abstract

In recent years, the research on optical remote sensing images has received greater and greater attention. Object detection, as one of the most challenging tasks in the area of remote sensing, has been remarkably promoted by convolutional neural network (CNN)-based methods like You Only Look Once (YOLO) and Faster R-CNN. However, due to the complexity of backgrounds and the distinctive object distribution, directly applying these general object detection methods to the remote sensing object detection usually renders poor performance. To tackle this problem, a highly efficient and robust framework based on YOLO is proposed. We devise and integrate VaryBlock to the architecture which effectively offsets some of the information loss caused by downsampling. In addition, some techniques are utilized to facilitate the performance and to avoid overfitting. Experimental results show that our proposed method can enormously improve the mean average precision by a large margin on the NWPU VHR-10 dataset.

## 1. Introduction

Artificial intelligence (AI) has attracted attention as a key for growth in developed countries and developing countries [1]. In the past few years, convolutional neural network (CNN)-based algorithms have played an important role in AI and achieved flourishing success for computer vision tasks [2,3,4]. Meanwhile, plenty of high resolution satellite and aerial images have boosted the research and investigations of remote sensing technology [5]. And with the continuous improvements of Unmanned Aerial Vehicle (UAV) technology, more and more UAVs are applied in environmental detection, resource detection, ecological research, surveying and mapping and military command [6,7,8,9]. As one of the most challenging problems in the area of computer vision, object detection, have witnessed the great power of convolutional neural networks. Various object detection algorithms based on convolutional neural networks have emerged in recent years [10,11,12,13,14], such as Faster R-CNN [15], YOLO [16], Single Shot MultiBox Detector (SSD) [17], RefineDet [18] and CornerNet [19]. They have gained outstanding performance in nature scene image datasets and have achieved breakthroughs on PASCAL VOC dataset and COCO dataset [20,21].

However, most of the current object detection algorithms’ performance on optical remote sensing imagery are barely satisfactory, due to the complicated characteristics of the optical remote sensing images—for example, some bridges and roads are too similar to be distinguished, have almost exactly the same appearances [6]. So far, there have been few studies on how to deal with the information loss caused by down-sampling. To tackle with this issue, Cheng-Yang Fu et al. [22] added contextual information by using deconvolution layer. Joseph Redmon et al. [16] brought upsample layer into the network architecture of YOLOv3, made it more accurate than their previous work YOLOv2 [23]. They all made a good suggestion to make up for the loss of information but still not delicate enough. Effective solutions are still scarce and current general object detection algorithms are yet incapable of retarding the information loss. Therefore, based on the excellent network structure of YOLO, we used YOLO’s structure as our backbone network. Designed a more specific and more well designed module, as continuation and reference of deconvolution and upsample layer. We propose a highly efficient and robust framework called VaryBlock, which can effectively remedy some of the information loss caused by down-sampling.

In this paper, we aim to study how to offset the information loss and damage caused by down-sampling. The main contributions of this paper are as follows:

1. A block named VaryBlock is proposed to treat the information loss and damage resulted from the downsample operation. When down-sampled, part of the image information will be discarded. Though, VaryBlock can effectively solve this problem.
2. A modified YOLO framework is designed, which succeeded in making better use of the semantic information of the original image without losing huge amounts of information. It not only enables raising the possibility that small objects could be detected, but also the other scales.
3. Some training trips that can improve the performance are presented and advanced data augmentation methods are used.

The remainder of this paper is organized as follows. In Section 2, previous works and some germane operations are presented. In Section 3, we describe the proposed method in detail. In Section 4 the experiment details and the utilized dataset is introduced. The comparison of our method with others and the relevant results are presented in Section 5. Finally, we conclude our research in Section 6.

## 2. Related Works

### 2.1. CNN-Based Object Detection Algorithm

Previous research has been a miraculous success with regard to optical remote sensing images object detection. Generally, the object detection algorithms can be divided into two categories— (a) one-stage object detection algorithms such as SSD, Deconvolutional Single Shot Detector (DSSD)  [22] and YOLOv3. (b) two-stage object detection algorithms such as SPPNet [24], Fast R-CNN [25], and Faster R-CNN.

Directly generating object classes probabilities and coordinates, the one-stage detection algorithms get rid of the stage of region proposal. Therefore, its detection speed is relatively fast. The core idea of SSD is to use convolution kernels to predict a series of default bounding boxes categories and coordinate shift on feature maps. In order to improve the mean Average Precision (AP) value of object detection, the prediction is made on the feature maps of different scales [26]. SSD directly predicts the coordinates of the bounding box and categories, exclusively generates proposal process. DSSD brings additional large scale context into the state-of-the-art general object detection, which associates Residual-101 [27] with SSD. Additionally, deconvolution module is added to enhance the characterization capability of shallow feature map. As an object detector with feature map upsampling and concatenation, YOLOv3 provides not only insights globally about the input image and all the objects in the picture, but achieves end-to-end training. Besides, the upsample layer is brought into the network architecture of YOLOv3.

The two-stage detection algorithms consider the detection problem in two stages. First, the region proposals are generated; and then the information from the images in the region proposals is classified. In a general way, location refinement is required in the classification step. SPPNet makes it possible to generate a fixed-length representation regardless of image size/scale. Fast R-CNN adopts the SPPNet method on the foundation of R-CNN, warps regions of interest (RoI) into one single layer using the RoI pooling. Faster R-CNN can be simply regarded as a combination of region proposal network (RPN) and Fast R-CNN. The regional proposal network comes up to generate proposal regions quickly. The main contribution of Faster R-CNN is that the RPN is designed to extract proposal regions and the region proposal extraction step is incorporated with the network, which significantly improves the detection speed.

All the methods mentioned above have made certain achievements to solve the detection problem. They tend to make better use of each feature map by all sorts of different ways such as feature map upsampling, feature maps fusion and anchors setting. However, they are still incapable of retarding the information loss. To alleviate the above-mentioned shortcomings, an object detection method based on YOLO is proposed and the proposed method can enormously raise the performance by a large margin on the utilized dataset.

### 2.2. Shortcut Connection

Assume the input of a certain neural network is *x* and the expected output is $H\left(x\right)$. However, $H\left(x\right)$ is difficult to learn; if we employ shortcut connections to the input *x*, then the goal we need to learn is $F\left(x\right)=H\left(x\right)-x$, instead of $H\left(x\right)$. So it is equivalent to changing the learning goal, no longer learning the complete output, but optimizing the difference between the solution $H\left(x\right)$ and the congruent map *x*, thus enabling a deeper network [28].

## 3. Details of Network Structure and Proposed Method

### 3.1. Overall Architecture

**Figure 1:**
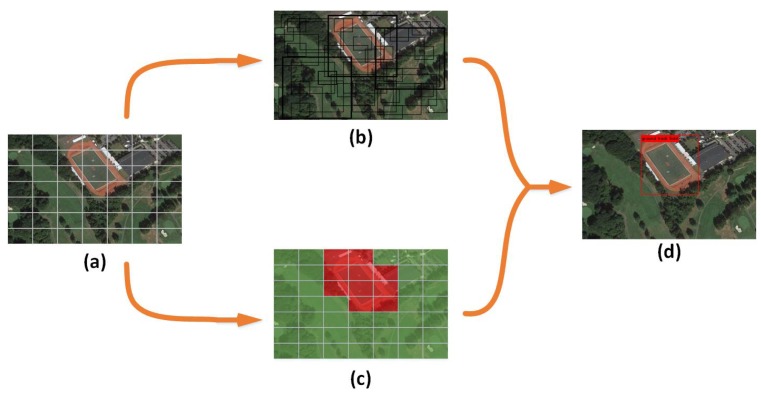
The overall flowchart of the proposed algorithm. The input image is divided into an equal-sized s×s grids (**a**), each grid includes three different scales and each scale owns three bounding boxes with different sizes (**b**,**c**) to predict the object (**d**).

In this section, we first present the overall flowchart of our algorithm. Then we introduce the details of VaryBlock, the architecture of both feature extraction model and detection model. We approach object detection task as a tensor-regression problem. A single neural network is designed and applied to the whole input image, which uses the input image an s×s grid to predict the object category and location directly and further simplifies both the training and the detecting process. The overall flowchart of the proposed algorithm is illustrated in Figure 1.

Our method mainly involves a feature extraction model using VaryBlock and detection model. Both of them are actually an integrated structure that has end-to-end characteristics. As these two models share the convolutional layers, object detection is unified in a deep neural network. This enables the possibility of end-to-end characteristics of the entire architecture. The initial convolutional layers of the neural network are in charge of extracting features from the input image. It alternately uses 1×1 convolutional layer to reduce the feature space from preceding layers [29]. The output of detection was a cluster of bounding boxes which include the confidence score and location information of the objects. Among these predicted boxes, we select the box with the highest confidence score.

**Figure 2:**
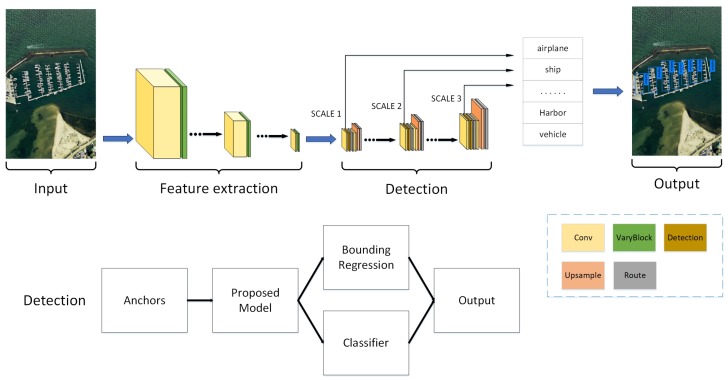
Framework of proposed method.

As is shown in Figure 2, during the detection process, anchors are generated to find out the possible object area on the input image across our proposed model. Then we employ regression and classification towards the obtained bounding box Finally, the position of the bounding box and class probabilities of the objects are treated as output.

### 3.2. VaryBlock

**Figure 3:**
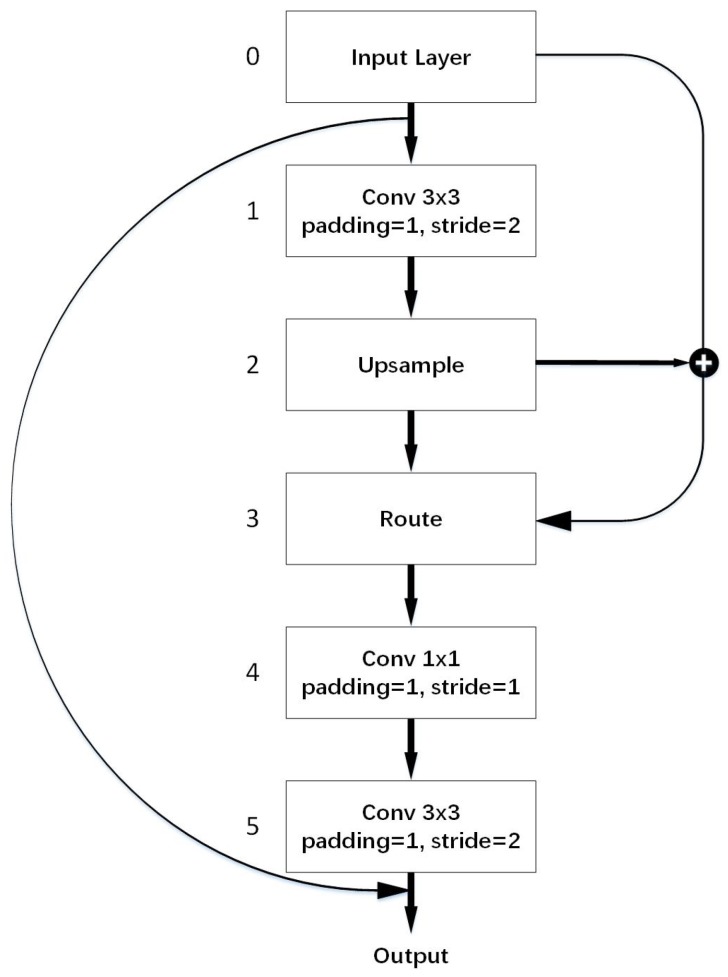
Architecture of VaryBlock, consisting of five layers exclusive of layer zero.

Small object detection requires a high resolution image, whereas most of the current general object detection algorithms cannot make effective use of abundant information from the original image. With the deepening of the network, a lot of underlying feature information or small object details often vanish, which has a great impact on the detection of small objects in remote sensing. Existing object detection algorithms rarely consider the information loss caused by down-sampling, which makes it difficult to cope with small object detection in geospatial remote sensing. To overcome this obstacle, we come up with VaryBlock. The architecture of the VaryBlock is shown in Figure 3. Inspired by the deconvolution and upsample layer, we propose VaryBlock. Instead of using down-sample layer crudely, we used VaryBlock as a substitute, which is precisely designed. It also provided a more specific solution to the problem of information loss. And ultimately through a convolutional layer, it moves to the next layer slowly. In order to enable the detection model network to utilize more microscopic information, we use VaryBlock in the front part of the overall structure. Route and Upsampling layers are used in VaryBlock to combine information from different feature map levels and gain as much semantic information as possible.

As is shown in Figure 3, the VaryBlock contains five layers. The zeroth layer is the input of VaryBlock. We regard it as a layer outside of VaryBlock.

The first layer is a convolutional layer, which contains multiple 3×3 convolutional kernels with a padding of 1 and a stride of 2. Namely, after passing through the Input Layer, the dimension of the feature map is reduced to 1/2 of the size of the input layer by downsampling in this layer. We can also see that, after using the convolutional kernel, 1/2 of the image information has been lost.

The second layer is the Upsample layer, which makes the height and width of the input feature maps 2 times larger while the number of channels has not changed.

**Figure 4:**
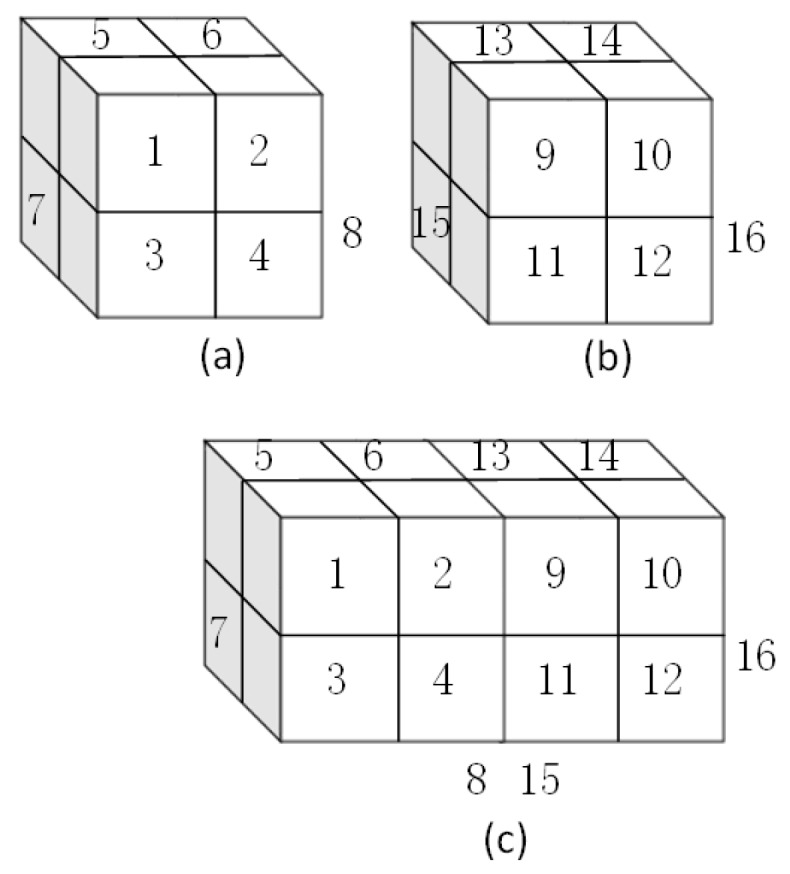
The route operation: (**a**) A = [[(1,2), (3,4)], [(5,6), (7,8)]];(**b**) B = [[(9,10), (11,12)], [(13,14), (15,16)]]; (**c**) C = [[(1,2,9,10), (3,4,11,12)], [(5,6,13,14), (7,8,15,16)]]. C is the route operation of A and B.

The third layer is the Route layer. The output of the Route layer is used to merge the Input Layer and the Upsample layer like a route sign pointing to the two layers we want to concatenate. Figure 4 shows the route operation in detail.

The fourth layer is a convolutional layer that includes 1×1 convolutional kernels with a padding of 1 and a stride of 1. This layer is not only used to amend the number of channels but to enable more feature combinations. Most simplistic explanations will be that 1×1 convolution leads to dimensionality reduction [29].

The fifth layer is also a convolutional layer with a kernel of size 3×3. Via this layer, the dimension of the feature maps is the same as that of the Input layer. After replacing VaryBlock with the general downsample layer, the convolutional neural network can remedy some information loss resulted from downsampling. Finally, the Input Layer and the fifth layer are connected by shortcut operation in order to train a deeper network. Finally, we employ the shortcut connection to link the output of the zeroth layer and the output of VaryBlock. The convolution operation and the shortcut connection are detailed in related works.

It should be noted that the number of filters in 1×1 and 3×3 convolution kernels inside the VaryBlock is 2 and 4 times the number of filters in the corresponding input layer, respectively.

### 3.3. Structure of the Proposed Method

#### 3.3.1. Feature Extraction Model

**Figure 5:**
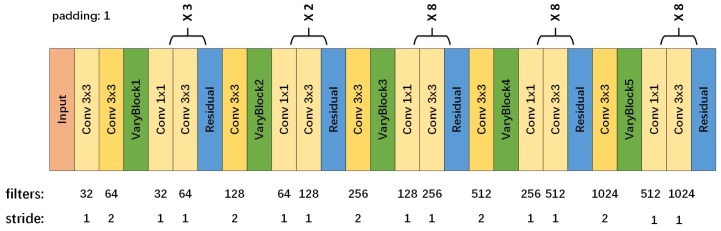
The structure of feature extraction model with a mixture of 1 × 1, 3 × 3 convolution layer, VaryBlock and Residual layer.

As is known above, VaryBlock provides a solution to make up for the deficiency of downsampling. It further enhances the performance of object detection. Consequently, we propose a feature extraction model to replace traditional downsampling with VaryBlock. We fed an input image through the fully convolutional network to build a rich feature representation of the original image. The model with a mixture of 1×1, 3×3 convolution layer, VaryBlock and Residual layer should lead to excellent performance in feature extraction. Taking the Residual layer into account, there were 106 layers in total. Moreover, it has a top-to-bottom architecture and incredible feature extraction capability so that it can learn a lot of feature information that the general object detection model is unable to learn. In fact, it is a critical role to elaborately design the feature extraction model for object detection. The structure of the feature extraction model is shown in Figure 5.

**Figure 6:**
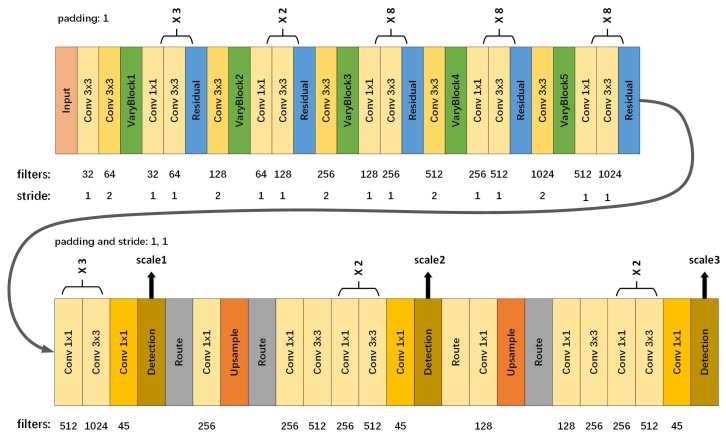
The overall structure of proposed method.

On the basis of YOLO, some improvements are made towards feature extraction network. As shown in Figure 6, four additional convolutional layers are added to the shallow neural network (the front part of the extraction model) in order to learn more about the underlying characteristics. The VaryBlocks are used to replace traditional downsampling layers in a bid to offset the image information loss. Compared with the general methods of object detection, our feature extraction model should have a better ability to extract features from the former layers.

#### 3.3.2. Detection Model

We directly use the detection part of YOLO as our backbone structure. Without huge modification, we only change the last route layer setting from (-1,11) to (-1,36) in order to fuse the underlying features instead of the middle layer features. Moreover, we alter the stride of the last Upsample layer from 2 to 4. In order to make small objects more easily to be detected, we use three different scales for detection. With 32 layers altogether, the detection model is composed of 1×1 convolution layers, 3×3 convolution layers, Unsampled layers and Detection layers. More specifically, three detection layers are included, corresponding to three scales—small-scale, medium-scale and large-scale. In particular, there is no Residual layer or varyblocks in the model. The output tensors of the detection model involve the information of predicted bounding boxes such as center coordinates, width, height, confidence score and class probabilities. The structure of the detection model is shown in Figure 6 (lower part). it should be noted that the left side is the input of the detection model and the output of the feature extraction model. More details can be found in Reference [16].

#### 3.3.3. Overall Network Structure

For the sake of understanding, we subjectively split the model into two parts in the above. However, it is an end-to-end fully convolutional neural network. In fact, the feature extraction model is followed by the detection model, which can be seen in Figure 6, where the number over parentheses indicates the repetition times of the small structure, which are enclosed by the braces. The order from the shallow to the deep network is from left to right and top to bottom in Figure 6.

As is shown in Figure 6, we integrate the feature extraction model and the detection model to achieve the end-to-end characteristic of our proposed algorithm and to further avoid the cumbersomeness of the algorithm. Therefore, our algorithm is easy to understand. The entire algorithm based on modified YOLO is detailed in Algorithm 1.

**Algorithm 1** Object detection algorithm based on modified-YOLO.**Input:** Image $x_{i}$ and Model $M_{Weights}$ **Output:** Prediction accuracy probability *P* of $x_{i}$ and the corresponding anchors position *C*
 1:Load the image $x_{i}$; 2:Load the proposed model weights $M_{Weights}$; 3:Generate several matrix vectors of different a priori anchors; 4:Put the matrix vectors as the input data of the whole algorithm; 5:Scan the grid by anchors and perform convolution operations to calculate the feature vector of the grid cells, which are located in the bounding box; 6:Apply the logistic regression and feature model $M_{Weights}$ to obtain the prediction accuracy probability *P* of image $x_{i}$; 7:Output the prediction accuracy probability *P* that is used to predict the class of $x_{i}$ and the corresponding coordinate information.


### 3.4. Data Augmentation

**Figure 7:**
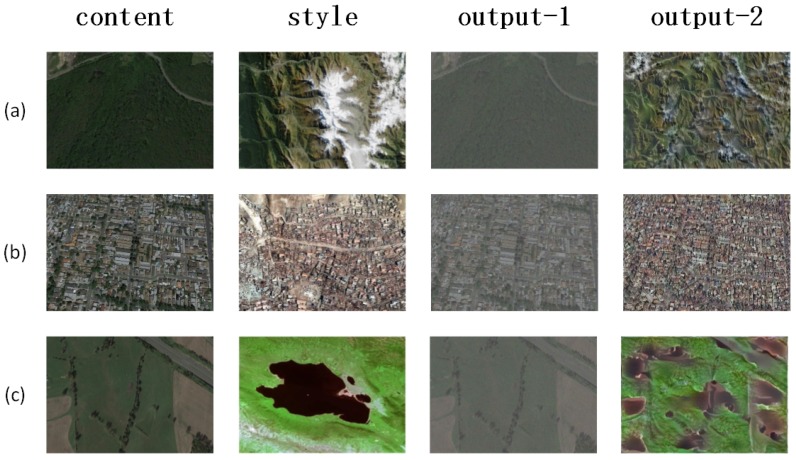
Negative samples after using cycleGAN. Given content and style, then generate the fake negative samples. output-1 and output-2 show two different fake samples. (**a**) Mountain (**b**) Town; (**c**) Swamp.

**Figure 8:**
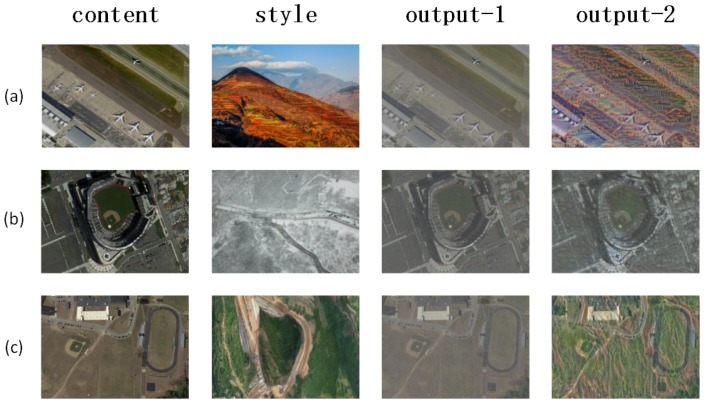
Positive samples after using cycleGAN. Given content and style, then generate the fake positive samples. output-1 and output-2 show two different fake samples. (**a**) Airport; (**b**) Baseball field; (**c**) Sports park.

Along with the appearance of Generative Adversarial Networks (GAN), GAN has excellent performance in various research fields and has derived many applications. In recent years, GAN has been widely used in data augmentation and object detection. It is already one of the most advanced data augmentation methods, so we use cycleGAN [30] to achieve style transfer, and then use traditional methods (rotation, cropping, etc.) to generate more data. We select some data from the result of generation, and the training set and the test set are expanded three times, respectively. The visual results can be seen in Figure 7 and Figure 8.

**Figure 9:**
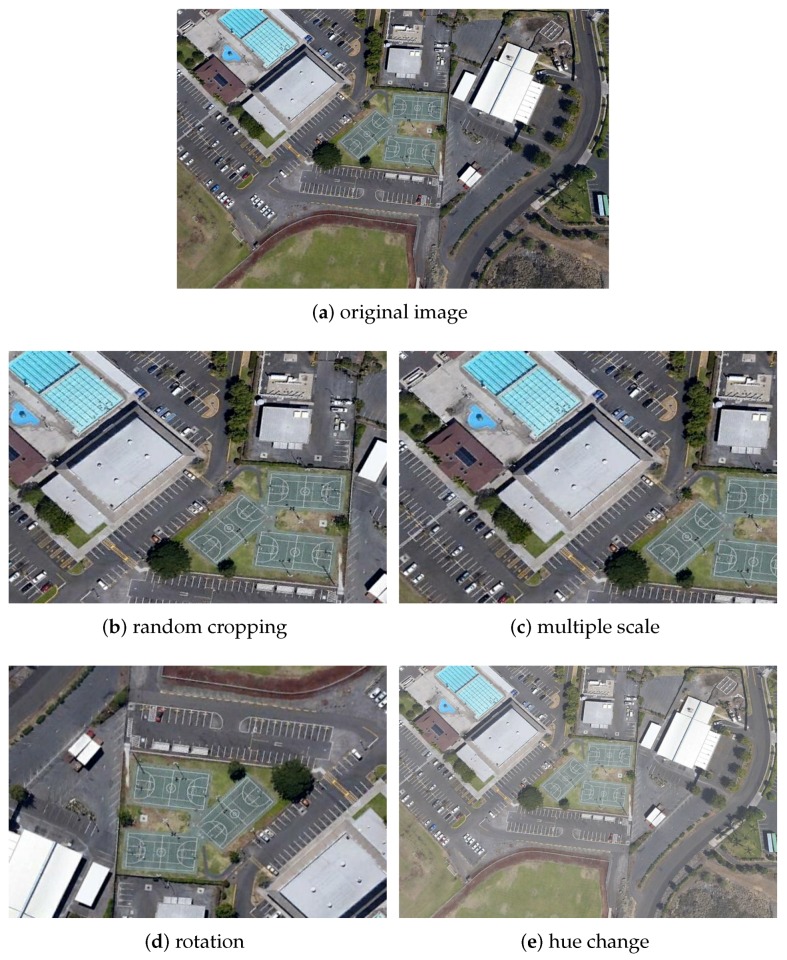

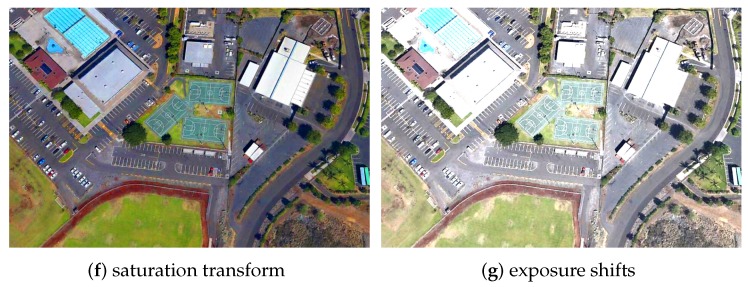
Example of Data Augmentation.

After using cycleGAN, traditional data augmentation methods have been adopted—random crop, multiple scale, rotation, change hue, saturation transform and shift exposure. More intuitionistic results are shown in Figure 9.

### 3.5. Generate Priori Anchors

In the original YOLOv3, anchors are obtained in the case of using the K-means clustering algorithm on the VOC dataset. However, the object sizes in the remote sensing image dataset are quite different from the 20-classes dataset which usually have relatively small size. It is not reasonable for some of the anchors if we directly use our utilized dataset to train the detection object. Therefore, we recalculate the anchors on the NWPU VHR-10 dataset to improve the detection rate of the anchors.

To solve the problem that K-means clustering algorithm is sensitive to the initial point selection, we use the K-means++ clustering algorithm [31] to obtain the priori anchors, which is detailed in Algorithm 2.

**Algorithm 2** generate priors anchors**Input:** Anchors size set *X* of objects in the dataset **Output:** The size of 9 different scale anchors set *Y*
 1:Choose one cluster center uniformly at random from the set *X*; 2:For each point *x* in *X*, compute D(x). The distance between *x* and the nearest center that has already been chosen; 3:Choose one new data point at random as a new center, using a weighted probability distribution where a point *x* is chosen with probability proportional to $D{\left(x\right)}^{2}$; 4:Repeat Step 2 and 3 until 9 centers have been chosen; 5:Employ standard K-means clustering on set *X*; 6:Output the set *Y*.


Before training, we select 9 anchors and 3 scales then evenly split the anchors across the scales and use the K-means++ clustering algorithm to determine the a priori anchors. The original anchor sizes used in YOLOv3 and anchor sizes after recalculation are shown in Table 1 and Table 2, respectively.

## 4. Experiments and Datasets

### 4.1. Datasets

In this section, the dataset that we used to evaluate the effectiveness of our proposed algorithm for remote sensing object detection is introduced. The NWPU VHR-10 dataset is an open satellite image object detection dataset which contains totally 800 very-high-resolution (VHR) optical remote sensing images. Within the dataset, there are 715 images acquired from Google Earth with a 0.5–2.0 m spatial resolution and 85 pan-sharpened color infrared images with a 0.08m spatial resolution. It has two sub datasets, a positive dataset with 650 images and a negative dataset with 150 images. In each image in the positive dataset there exists at least one target to be detected and all images in the negative dataset do not have any target of the given classes to be detected [32]. Meanwhile, we simply and randomly divide the positive dataset into 70% for training and 30% for testing, respectively, namely 560 images for training, 140 images for testing.

**Figure 10:**
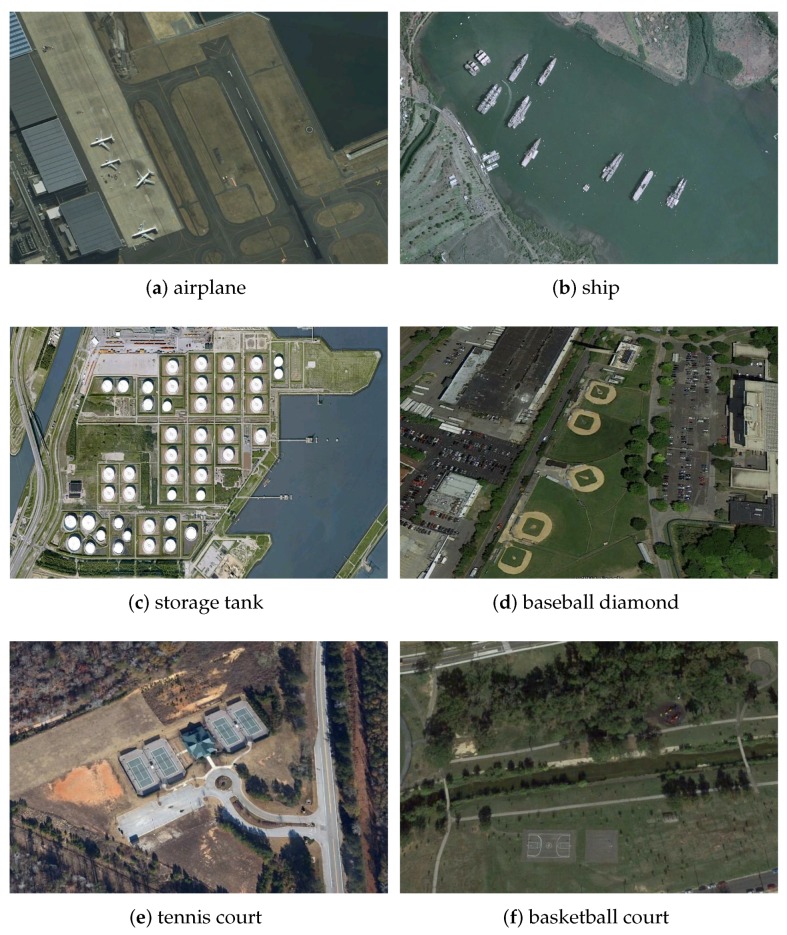

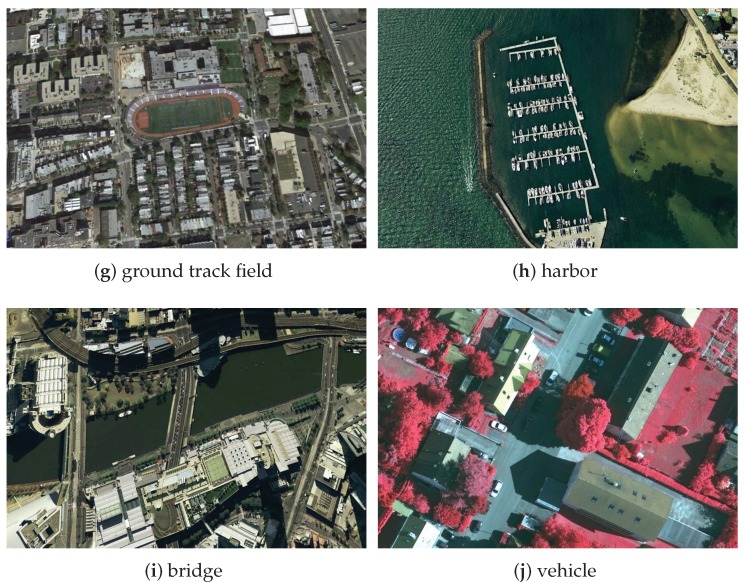
Some examples of the NWPU VHR-10 dataset.

The utilized dataset contains the following 10 categories—airplanes, ships, storage tanks, baseball diamonds, tennis courts, basketball courts, ground track fields, harbors, bridges and vehicles. Some samples of the NWPU VHR-10 dataset are shown in Figure 10.

**Figure 11:**
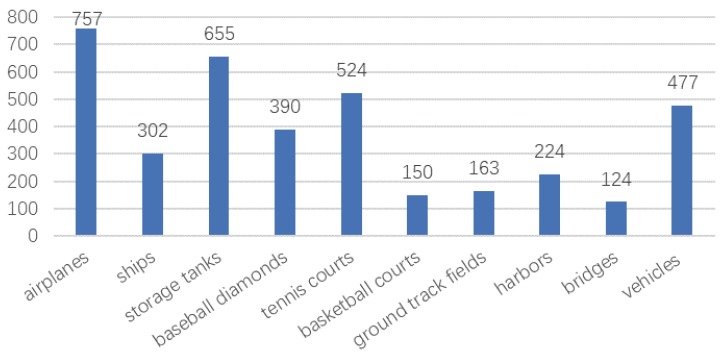
Histogram of the number of classes in the NWPU VHR-10 dataset.

As for the positive image set, 757 airplanes, 302 ships, 655 storage tanks, 390 baseball diamonds, 524 tennis courts, 150 basketball courts, 163 ground track fields, 224 harbors, 124 bridges and 477 vehicles were manually annotated with bounding boxes and utilized as the ground truth [32]. The distribution of 10 classes in the NWPU VHR-10 dataset is shown in Figure 11.

### 4.2. Experimental Metric

To trade off the widely used precision and recall, the AP computes the average value of the precision over the interval from recall = 0 to recall = 1, that is, the area under the Precision Recall Curve (PRC). Hence, the higher the AP, the better the performance [33]. In addition, mean AP (mAP) computes the average value of all the AP values for the ten classes in the dataset. The precision and recall metrics can be formulated as follows with TP, TF, FP and FN standing for true positives true negative, false positives and false negative, respectively:

(1) $$\begin{array}{ccc} \mathrm{Precision} & = & \frac{\mathrm{TP}}{\left(\mathrm{TP}+\mathrm{FP}\right)} \end{array}$$

(2) $$\begin{array}{ccc} \mathrm{Recall} & = & \frac{\mathrm{TP}}{\left(\mathrm{TP}+\mathrm{FN}\right)} \end{array}$$

If the area of intersection over union (IoU) between the predicted bounding box and ground truth surpasses 0.5, it shall be deemed a true positive; otherwise, it shall be deemed a false positive. In addition, if the overlap ratio between multiple detections and the ground truth are larger than 0.5 simultaneously, only the bounding box with the largest area IoU is considered a TP, others are considered a FP [34]. Note that in the experiment, the IoU threshold is fixed at 0.7.

### 4.3. Implementation Details

In this section, our implementation details and computational platform are listed. We select images randomly from the DOTA dataset and the Open Images dataset for pre-training using SGD (Stochastic Gradient Descent). And then load the resulting weight files into the model as pre-training weights. Our experiments were performed on a Linux PC with an Intel i7-4790 CPU, 16 GB DDR3 and an Nvidia GeForce GTX1080Ti with 11 GB of memory. Besides, during the experiment, we followed the niche framework - Darknet, made some modifications on it and added the VaryBlock module to the network. Some training itineraries and advanced data enhancement methods were also participated on this basis.

To make the proposed detector more robust to diverse input object with multiple sizes and shapes, each training image is randomly sampled into 70% for training and 30% for testing. In the training phase we train a total of 30,000 iterations with a basic learning, momentum and weight decay coefficients of 0.001, 0.9, and 0.0005, respectively We use stochastic gradient descent with a batch size of 64 and a subdivision size of 32. Namely, 64 samples are randomly selected from all the training sets for each iteration to participate in the training; Then, all the batch samples are divided into 32 times and sent to the network for participating training in order to reduce the pressure of memory occupation. To prevent overfitting, data augmentation methods are used during the training period: cropping randomly, rotation, hue change, saturation transform, exposure shifts. For the input image, we adopt the size of 480×480 pixels and the number of channels is 3 which enhance the performance of small object detection. Besides, all the results in the experiment are the averages of multiple random experiments(10 times).

## 5. Results

**Figure 12:**
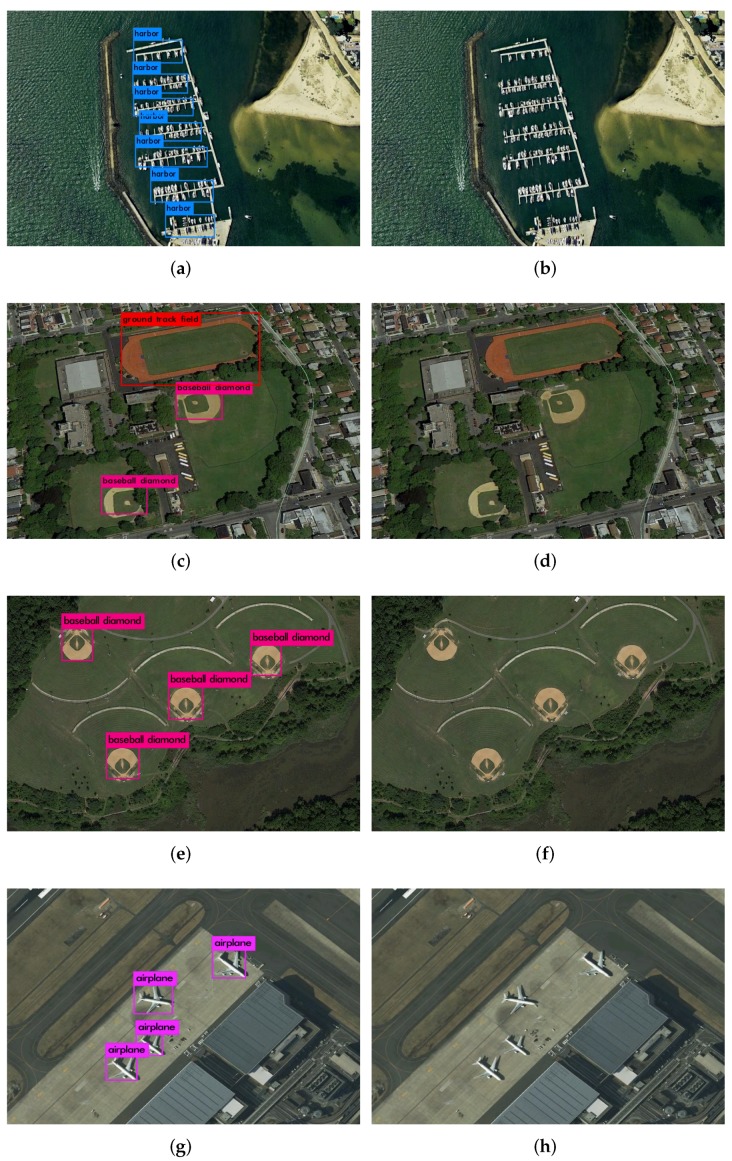
Detection results using our method. The first column are results of our method; the second column are the relevant original image. (**a**) Harbors detected from scene (**b**); (**c**) Ground track field and baseball diamound detected from scene (**d**); (**e**) Baseball diamound detected from scene (**f**); (**g**) Airplane detected from scene (**h**).

In this section, we first present visualized detection results across our method. To make a fair comparison, the dataset is randomly split into 70% for training, and 30% for testing. Some examples of the detection results are shown in Figure 12.

To quantitatively validate the effectiveness of our proposed method, experiments are carried out by testing the mean AP values on a 10-classes NWPU VHR-10 dataset. Comparing with diverse methods, including Transferred CNN [5], YOLOv2, RICNN [5], SSD, R-P-Faster R-CNN [35], Faster R-CNN and the baseline YOLOv3, relevant experimental results show the superiority of our proposed algorithm. In addition, the results of other methods all come out of the previous papers. The results of the various methods on NWPU VHR-10 dataset are shown in Table 3. We used a single GPU during the experiment, the FPS was between 20–25, which can basically meet the needs of real-time. If multiple high-performance GPUs are used, the computing time will be significantly improved.

Compared with other object detection methods, the results indicate that the method we proposed obtains the best mAP value of 92.0% among all and achieves the highest mAP value for most classes. In particular, the mAP values of objects that covers most sizes, increase dramatically. It illustrates the magnificent performance of our methods for remote sensing object detection.

**Figure 13:**
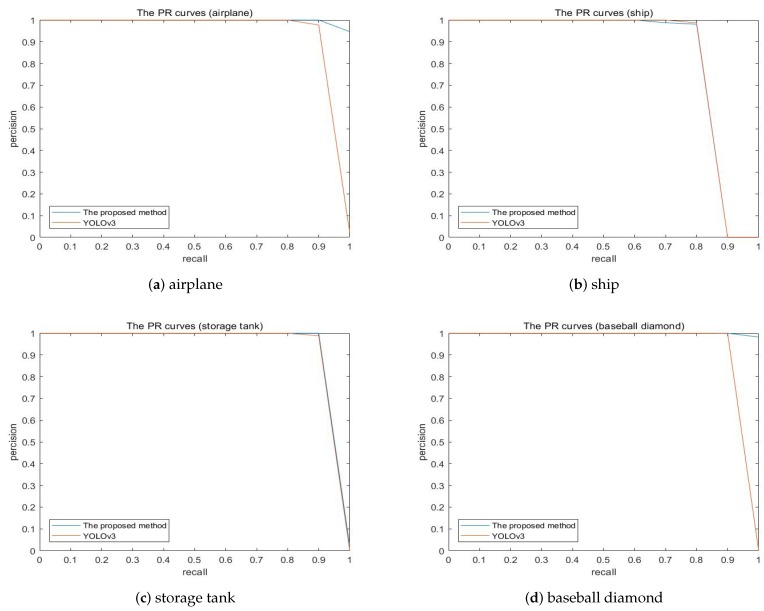

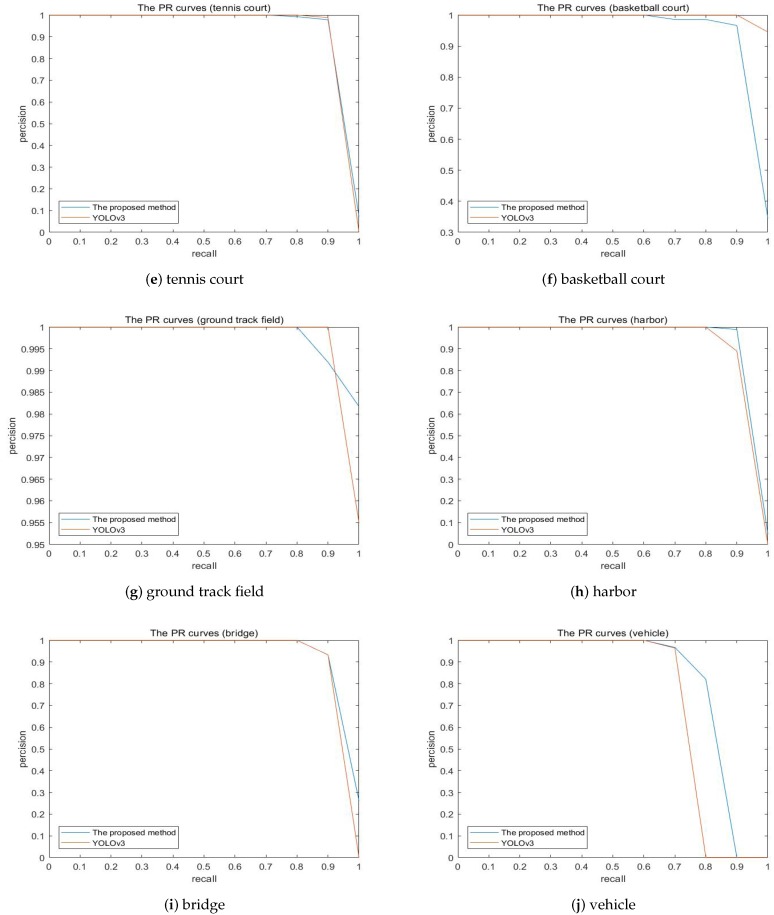
The precision-recall curves of the results using YOLOv3 and proposed method on the NWPU VHR-10 dataset.

**Figure 14:**
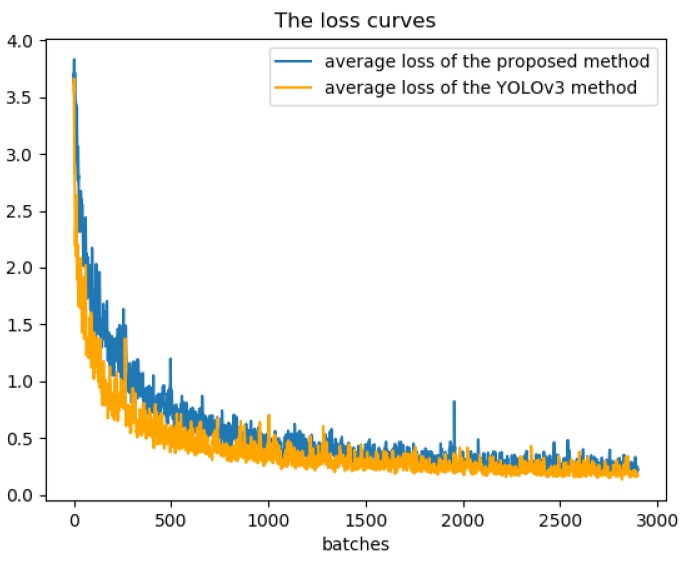
The training loss curves of our proposed method and YOLOv3.

Compare our proposed algorithm with the baseline YOLOv3, the AP values of nine classes are increased, including airplane (0.909 to 0.995), storage tank (0.908 to 0.911), baseball diamond (0.909 to 0.998), tennis court (0.908 to 0.913), ground track field (0.9959 to 0.9976), harbor (0.899 to 0.912), bridge (0.903 to 0.927) and vehicle (0.724 to 0.799). Obviously, all classes have different levels of lift which can verify the robustness of our model. Meanwhile, the baseball diamond obtains the best performance, in comparison to other objects adopting the proposed method. Moreover, in comparison with the baseline, our proposed method behave little worse at two sorts. This may be caused by a variety of factors. However, the most likely reason may be that the images generated by cycleGAN have randomness, and different noises may result in slightly difference experimental results. In the future work, we will do further research on this case. Figure 13 show the precision-recall curves of the results using our proposed method and YOLOv3. The loss curves of our proposed method and YOLOv3 are illustrated in Figure 14.

Figure 14 shows that compared with the original YOLOv3 algorithm, our model has a restively low and steady loss value, during the entire training process. It also indicates that our method can effectively reduce the loss value of the training process and make model training more efficient.

## 6. Conclusions

In this paper, we propose an end-to-end object detection method for remote sensing images using modified YOLO framework Considering the information loss caused by down-sampling and the unsatisfactory efficiency of the current general object detection methods for HSR remote sensing imagery, we design a layer named VaryBlock, and integrate it into the feature extraction model. In addition, data augmentation is used to solve the problem of insufficient remote sensing image data. Meanwhile, we directly take the detection sector of YOLOv3 algorithm as our detection model due to its high efficiency. The experimental results demonstrate that the method we propose can accurately detect diverse types of objects in complex backgrounds, and has exhibited magnificent performance on the NWPU VHR-10 dataset. Nonetheless, there is still plenty of room for the improvement in our work. There will be valuable in using any other useful methods such as transfer learning or combining context information in our method, which will be the priority of our future research studies.

## Figures and Tables

**Table 1:** The anchors size of original YOLOv3.

**width**	10	16	33	30	62	59	116	156	373
**height**	13	30	23	61	45	119	90	198	326

**Table 2:** The anchors size after recalculation.

**width**	18	25	38	41	87	55	95	165	122
**height**	27	49	34	64	59	107	203	140	260

**Table 3:** The AP values of the eight object detection methods.

	Transferred CNN	YOLOv2	RICNN with Fine-Tuning	SSD	R-P-Faster R-CNN (Single VGG16)	Faster R-CNN	YOLOv3	OURS
Airplane	0.661	0.733	0.884	0.957	0.904	0.946	0.909	0.995
Ship	0.569	0.749	0.773	0.829	0.750	0.823	0.817	0.816
Storage tank	0.843	0.344	0.853	0.856	0.444	0.653	0.908	0.911
Baseball diamond	0.816	0.889	0.881	0.966	0.899	0.955	0.909	0.998
Tennis court	0.350	0.291	0.408	0.821	0.790	0.819	0.908	0.913
Basketball court	0.459	0.276	0.585	0.860	0.776	0.897	0.995	0.935
Ground track field	0.800	0.988	0.867	0.582	0.877	0.924	0.9959	0.9976
Harbor	0.620	0.754	0.686	0.548	0.791	0.724	0.899	0.912
Bridge	0.423	0.518	0.615	0.419	0.682	0.575	0.903	0.927
Vehicle	0.429	0.513	0.711	0.756	0.732	0.778	0.724	0.799
mean AP	0.597	0.605	0.726	0.759	0.765	0.809	0.897	0.920
